# Cancer Detection and Surveillance Pathways with Abbreviated MRI in the Prostate, Pancreas, and Liver: A Risk-Stratified Narrative Review

**DOI:** 10.3390/curroncol33060306

**Published:** 2026-05-24

**Authors:** Hannah Brown, Sharon E. Clarke

**Affiliations:** 1 School of Medicine, Queen’s University, Kingston, ON K7L 3N6, Canada; 2 Department of Radiology, Dalhousie University, Halifax, NS B3H 2Y9, Canada; sharon.clarke@dal.ca; 3 Diagnostic Imaging, Nova Scotia Health Authority, Halifax, NS B3H 2Y9, Canada

**Keywords:** narrative review, abbreviated MRI, cancer detection and surveillance, biparametric MRI, prostate cancer, hepatocellular carcinoma surveillance, pancreatic cystic lesions

## Abstract

Magnetic resonance imaging (MRI) plays an essential role in cancer detection and surveillance, yet complete MRI (C-MRI) protocols are lengthy, resource-intensive, and may limit access within population-level cancer care pathways. Abbreviated MRI (A-MRI) protocols have emerged as a streamlined alternative that may preserve diagnostic performance while reducing examination time, cost, and IV contrast exposure. We conducted a narrative review of studies published between 2015 and 2025 evaluating A-MRI in prostate cancer detection and surveillance, pancreatic cystic lesion surveillance, and hepatocellular carcinoma (HCC) surveillance. Evidence was synthesized qualitatively with emphasis on potential advantages, limitations, guideline positions, and evidence gaps. Across the organ systems assessed, A-MRI may demonstrate diagnostic performance comparable to C-MRI in selected clinical contexts. Biparametric MRI has demonstrated noninferior detection of clinically significant prostate cancer; non-contrast A-MRI may be considered for the surveillance of selected low-risk pancreatic cystic lesions, and A-MRI may offer higher sensitivity than ultrasound for HCC surveillance in patients with suboptimal ultrasound visualization. Heterogeneity in the available literature and limited prospective outcome data remain important limitations. Overall, A-MRI represents a complementary, risk-adapted strategy rather than a universal replacement for C-MRI, and further prospective studies are needed to define optimal patient selection and long-term oncologic benefit.

## Simple Summary

Magnetic resonance imaging is widely used to detect and monitor cancer, but standard examinations can be long, costly, and difficult to access. Shortened “abbreviated” magnetic resonance imaging protocols have been developed to reduce scan time while maintaining diagnostic accuracy. This review examines the role of abbreviated magnetic resonance imaging in prostate cancer detection and surveillance, pancreatic cyst surveillance, and liver cancer surveillance. Across these settings, abbreviated protocols may perform similarly to full examinations in selected patients and may improve access to imaging services. However, evidence remains limited in some areas, such as how studies were conducted and limited research following patients forward over time to confirm long-term outcomes. A risk-adapted approach, which involves using abbreviated imaging in appropriate clinical contexts while reserving full examinations for higher-risk situations, may help improve efficiency without compromising patient care. Further prospective studies are needed to determine how abbreviated imaging can be safely and effectively integrated into cancer care pathways.

## 1. Introduction

MRI plays a central role in contemporary cancer care, supporting early detection, risk stratification, biopsy guidance, and longitudinal surveillance [1,2]. However, complete MRI (C-MRI) examinations are time- and resource-intensive, which may constrain access, delay diagnosis, and limit integration within population-level screening and surveillance programs [3]. These challenges are especially relevant in clinical settings where MRI is indicated for a large number of patients or is used frequently or repeatedly. Among cancer care pathways with high MRI utilization, those involving the prostate, pancreas, and liver are particularly affected.

The National Comprehensive Cancer Network (NCCN) Prostate Cancer Early Detection Guideline provides a category 1 recommendation for the use of multiparametric MRI prior to biopsy in patients with elevated prostate-specific antigen being evaluated for prostate cancer [4]. Multiparametric MRI also serves as the primary imaging modality during active surveillance, with the NCCN recommending confirmatory MRI within 6 to 24 months of diagnostic biopsy, followed by repeat MRI no more often than 12 months thereafter, unless clinically indicated [4,5,6]. Together, these indications have contributed to increased demand for prostate MRI in an already resource-constrained imaging environment [6]. In the pancreas, cystic lesions are a common incidental finding, with a prevalence of approximately 6–16% on cross-sectional imaging [7,8]. These lesions require serial MRI follow-up to assess for the development of neoplastic features, contributing to a growing surveillance burden [7]. While guidelines are mixed in their recommendations for the duration of time needed for surveillance, the Fukuoka 2017, American College of Gastroenterology 2018, and European 2018 guidelines recommend indefinite or prolonged surveillance of presumed mucinous pancreatic cystic lesions [7,9,10]. In the liver, the American Association for the Study of Liver Diseases (AASLD) recommends that hepatocellular carcinoma (HCC) surveillance be performed in at-risk patients approximately every six months [11]. Although ultrasound with AFP remains the recommended first-line modality, visualization is suboptimal in approximately 20–50% of patients, depending on the population studied, prompting consideration of alternative imaging modalities, including MRI, for surveillance [11,12,13]. 

In this context, increasing demand for MRI highlights the need for imaging approaches that are more accessible and sustainable. Abbreviated MRI (A-MRI) protocols aim to improve efficiency by focusing on the sequences considered most diagnostically relevant, while omitting those that provide only incremental value [3]. Potential advantages include reduced examination time, lower cost and decreased IV contrast exposure [3]. In appropriately selected populations, A-MRI may improve access to MRI without compromising the ability to detect clinically significant disease.

This narrative review examines the evolving role of A-MRI in prostate cancer detection and surveillance, pancreatic cystic lesion surveillance, and hepatocellular carcinoma surveillance. These organ systems were selected because they represent high-volume clinical scenarios where MRI is used either for population-level detection (prostate) or longitudinal surveillance (pancreas and liver), making them particularly relevant for evaluating efficiency-driven imaging strategies such as A-MRI. The review focuses on the potential advantages, limitations, guideline recommendations, and key considerations for clinical adoption and broader integration into cancer care pathways. Although artificial intelligence-based methods that accelerate image acquisition at the sequence level are likely to play an increasingly important role in MRI efficiency, this topic is beyond the scope of this review.

## 2. Methods

Relevant literature was identified with the assistance of Sandra Halliday, Health Sciences Librarian at Queen’s University, using structured searches of Ovid MEDLINE and Ovid Embase with combinations of keywords and subject headings for each organ. Search terms were grouped into four domains: MRI technique, disease entity, abbreviated imaging approaches, and diagnostic/surveillance outcomes. Full search strategies are provided in the Supplementary Material in Table S2. Titles and abstracts were screened using Covidence. Reference lists of selected articles and key review articles were examined to identify additional relevant studies. Original research articles, consensus statements, and review articles were eligible if they were published between 2015 and 2025, focused on adults (≥18 years), and discussed A-MRI, C-MRI, or both. Non-English studies were excluded.

In keeping with narrative review best practices, evidence was synthesized qualitatively, with emphasis on potential advantages, limitations, guideline recommendations and gaps in the literature. A narrative review checklist was used to ensure methodological rigor and completeness. Supplementary Table S1 summarizes the checklist criteria that guided the review process.

## 3. Discussion of Abbreviated MRI Protocols

### 3.1. Prostate

#### 3.1.1. Background

Prostate cancer (PCa) is the second most common cancer in men, with over 1.4 million new cases reported in 2020 [14]. When detected early, the five-year survival approaches 100%, underscoring the importance of timely diagnosis [15]. In 2019, the American Urological Association and Society of Abdominal Radiology jointly endorsed multiparametric MRI (mpMRI) for all men being considered for biopsy, introducing important implications for imaging capacity and access within prostate cancer diagnostic pathways [16]. 

The Prostate Imaging Reporting and Data System (PI-RADS) is a standardized scoring system used to guide the interpretation of prostate MRI and assess the likelihood of clinically significant prostate cancer (csPCa) [17]. Scores range from 1 (clinically significant cancer is highly unlikely to be present) to 5 (clinically significant cancer is highly likely to be present) and help clinicians risk stratify and determine the need for biopsy. Within PI-RADS v2.1, the current version of the framework, mpMRI includes axial T2-weighted imaging (T2WI) in the axial or oblique axial plane plus at least one other orthogonal plane, diffusion weighted imaging (DWI), and dynamic contrast-enhanced (DCE) imaging. DCE is a component of mpMRI but has a reduced role in lesion characterization for treatment-naïve patients; it is of relevance in the peripheral zone (PZ) for PI-RADS 3 lesions but has no influence on PI-RADS scores for lesions in the transition zone. DCE serves as a modifier, upgrading PZ lesions from PI-RADS category 3 to 4 in the presence of early or contemporaneous focal enhancement, which increases the likelihood of csPCa and may prompt biopsy [18].

Consequently, biparametric MRI (bpMRI), which omits DCE and includes T2WI and DWI, has gained interest. BpMRI can reduce exam time from approximately 40 to 15 min and simplifies workflow by eliminating the need for venous access and IV contrast monitoring [19].

#### 3.1.2. Potential Advantages of Abbreviated Prostate MRI Protocols

There is some evidence supporting comparable diagnostic performance between bpMRI and mpMRI for detecting csPCa. Alabousi et al. (meta-analysis, *n* = 31) reported no significant difference in diagnostic test accuracy between mpMRI and bpMRI for detection of prostate cancer in treatment-naïve patients The pooled sensitivity was 86% for mpMRI vs. 90% for bpMRI and specificity was 73% for mpMRI vs. 70% for bpMRI [20,21]. Summary receiver operating characteristic curves were comparable for mpMRI (0.87) and bpMRI (0.90). However, study heterogeneity warrants cautious interpretation of the results. Of the included studies, 25/31 had unclear or high risk of bias and subgroup analysis found multiple sources of heterogeneity including study design, diagnostic test parameters, technical MRI parameters, and pooling of direct vs. indirect comparison studies. Additionally, most studies were retrospective, and several included bpMRI studies did not define the PI-RADS threshold for a positive test.

The PRIME study, a prospective, multicenter, within-patient, noninferiority trial of 490 biopsy-naive men, reported that bpMRI was noninferior to mpMRI for detection of Gleason Grade Group 2 or higher prostate cancer [19]. Of 490 men, csPCa was detected in 29.2% with bpMRI and 29.6% with mpMRI. Detection of clinically insignificant cancer was not statistically different between bpMRI and mpMRI. This study found that DCE changed treatment eligibility decisions in 4.3% of cases [95% CI, 2.7–6.5%] [19]. Wagner and Samji (retrospective, *n* = 2742) reported that 3.2% (87/2742) of the total number of mpMRI cases were upgraded to PI-RADS 4 lesions by DCE imaging [22].

A single-center retrospective study showed no difference in positive predictive value (PPV) between bpMRI- and mpMRI-directed TRUS-guided targeted biopsy for any cancer (55% and 56%, respectively) or csPCa (34% for both bpMRI and mpMRI) when adjusted for number of lesions and PI-RADS category [23]. This study was performed at an academic center, and results may not translate to other practice settings. 

Defining suitable reference standards for MRI examinations with true-negative findings and hence evaluating negative predictive value (NPV) is challenging. The BIDOC study, a single-institutional, paired, prospective cohort study of biopsy-naive men with clinical suspicion of prostate cancer who underwent both bpMRI-targeted and standard transrectal ultrasound-guided biopsies, low-suspicion bpMRI had a high negative predictive value (97%) in ruling out significant prostate cancer on confirmatory biopsies [24]. It is noted, however, that a low-suspicion bpMRI did not unequivocally rule out any prostate cancer. This study also used only one experienced reader and two experienced TRUS biopsy operators; less experienced readers and operators might not achieve the same diagnostic yield.

Salinas-Miranda et al. (meta-analysis, *n* = 18) in treatment-naïve patients found no difference in NPV for csPCa (92% for mpMRI and for 92% bpMRI) [21]. It was noted that 15 of the 18 studies evaluated simulated bpMRI examinations generated from mpMRI examinations by removing DCE images from the investigational interpretations. This design does not reflect the real-world clinical practice for MRI reporting or clinical decision making. Furthermore, no study randomized patients to undergo either bpMRI or mpMRI.

One potential strategy within a bpMRI framework is a system where patients with a PI-RADS 3 lesion in the PZ are recalled for IV contrast administration. This approach has demonstrated low recall rates. Rehman et al. (retrospective, *n* = 909) reported a recall rate of 5.7%, with 78.8% of recalls for PZ PI-RADS 3 lesions [25]. Brown et al. reported a similar 6.6% recall rate, of which 91.7% were recalled for PI-RADS 3 lesions [26]. Rehman et al. estimated cost savings of $68,000 USD and over 200 h of scan time over the study period [25] in the context of a single-payer Canadian health care system. These low recall rates suggest that a selective, stepwise approach to IV contrast administration may preserve diagnostic accuracy while maintaining workflow efficiency but need to be replicated in a variety of practice settings prior to consideration of wide-spread adoption.

#### 3.1.3. Limitations of Abbreviated Prostate Protocols

The omission of DCE removes a sequence that can provide important information in certain clinical scenarios; for example, DCE is useful for risk stratification of PIRADS 3 lesions. A study by Druskin et al. showed that the rate of csPC in PZ PI-RADS 3 lesions upgraded to PI-RADS 4 via positive DCE (termed PI-RADS 3 + 1) is higher than PI-RADS 3 but lower than PI-RADS 4 and this was most apparent in patients with a prior negative systematic prostate biopsy [27]. Greer et al. found lesions that are classified as category 3 by DWI and are DCE positive have a 68% chance of cancer detection, compared to 40% chance of cancer detection in category 3 lesions that are DCE negative [28]. Twilt et al. reported that 3% of PI-RADS 3 lesions were upgraded to PI-RADS 4 with DCE; however, 29% of those had csPCa [29]. This highlights the substantive impact DCE could have on csPCa diagnoses depending upon the biopsy threshold used. DCE is also important when T2WI and/or DWI are of reduced quality due to motion or susceptibility artifacts from rectal gas or hip implant. DCE is critical for interpretation in MRIs of previously treated glands and in evaluating for recurrence post prostatectomy using the Prostate Imaging for Recurrence Reporting System [30].

Reader experience is an important and debated factor. Some authors suggest that DCE may serve as a supportive tool for junior radiologists; however, evidence is mixed. Twilt et al. (international, paired, noninferiority confirmatory observer study, *n* = 400) demonstrated that bpMRI was noninferior to mpMRI for detecting csPCa, with nearly identical AUROC values (0.853 vs. 0.859) and noninferior sensitivity and specificity at PI-RADS ≥ 3 [29]. Subgroup analysis revealed minimal incremental benefit of DCE for non-expert readers [29]. 

A retrospective study by Gatti et al. (*n* = 68) demonstrated that DCE significantly improved performance among less-experienced readers. AUC for experienced readers was not significantly different between mpMRI and bpMRI (86% vs. 82%, respectively) but was significantly different for junior radiologists (82% vs. 52%) and residents (64% vs. 45%) [31]. This study is limited by retrospective design, small number of samples (45 cases of PCa and 23 controls) and small number of readers (two experienced staff radiologists, two junior staff radiologists, and two residents); therefore, the results cannot be generally applied to other practice settings. Implementation of A-MRI strategies will need to be tailored to local expertise and clinical workflow [31]. 

#### 3.1.4. Guidelines–Prostate MRI Protocols

Current guidelines do not specifically endorse bpMRI for the diagnosis or surveillance of prostate lesions. The PI-RADS Steering Committee acknowledges the potential benefits of bpMRI but advises that additional multicenter prospective studies including multiple readers be performed before explicitly supporting the omission of DCE in the assessment of treatment-naive prostate patients [32]. As such, the PI-RADS Steering Committee continues to recommend mpMRI as the standard approach, with bpMRI considered acceptable only in select clinical scenarios. Similarly, a joint panel of clinicians from the American Urological Association and Society of Abdominal Radiology recognizes both the advantages and limitations of DCE and encourages comparable prospective evidence before formal recommendations can be given [33]. The Canadian Association of Radiologists recommends mpMRI as the standard of care; however, given anticipated resource pressures, bpMRI can be performed at the discretion of the radiologist in centers that have demonstrated local bpMRI performance similar to mpMRI [34]. 

#### 3.1.5. Discussion of Abbreviated Prostate MRI Protocols

The current literature on bpMRI is heterogeneous and largely retrospective, with biopsy decisions often made based on mpMRI that included DCE. Study design based on simulated bpMRI examinations is a consistent limitation of the available literature on bpMRI [35]. Prospective studies in which biopsy decisions are guided solely by bpMRI findings in biopsy-naive and prior negative biopsy populations, as well as randomized trials with longitudinal follow-up, are needed. Methodologic variability further complicates comparison across studies. Definitions of csPC vary, with some studies using csPCa defined as a tumor with a Gleason score ≥ 7 and/or a volume ≥ 0.5 cm^3^ and/or extra-prostatic invasion [36] as per PI-RADS 2.1 [17] and International Society of Urological Pathology grade ≥ 2 [23] or as any core with high-grade prostate cancer (Gleason score greater than or equal to 7 (4 + 3) or maximum cancerous core length greater than 50% of Gleason score 7 (3 + 4) prostate cancer [24]. With respect to DCE, Belue et al. reported that only 51% of 41 studies reported csPCa prevalence among PI-RADS 3 lesions, and no studies stratified the benefit of DCE by MRI quality, while nearly half excluded cases due to technical limitations [37]. Differences in study design also influence reported diagnostic performance. Alabousi et al. reported higher specificity in prospective bpMRI studies 85% compared to retrospective bpMRI studies 57% [20]. Furthermore, most major trials and meta-analyses focus on biopsy-naive men [19,38]. 

Reference standards for evaluation of performance metrics vary across studies, including systematic biopsy, targeted biopsy, prostatectomy specimens and clinical follow-up [35]. Evaluation of true NPV is difficult because transperineal/saturation biopsies are performed less frequently and patients with negative MRIs may forgo biopsy; for example, in the PRIME study, subjects with an MRI not suggestive of cs PCa and a PSA density less than 0.15 ng/mL/mL did not undergo prostate biopsy [19]. Evidence of differences in clinical indications over time has been observed, reflecting a shift toward performing MRI prior to biopsy in biopsy-naive men [23].

DCE offers value in settings with limited reader experience, poor image quality, and for further evaluation of PI-RADS 3 PZ lesions and in previously treated glands. However, current indications for prostate MRI involve large and growing patient populations and increase pressure for timely access to MRI. In such contexts, bpMRI may provide a pragmatic approach where improvements in access and efficiency outweigh the benefit of DCE. Further research into specific populations and settings in which consideration of a bpMRI is warranted is required prior to widespread adoption. 

### 3.2. Pancreas

#### 3.2.1. Background

Pancreatic cystic lesions (PCLs) are increasingly detected due to the widespread use of imaging [7]. While most PCLs are benign, intraductal papillary mucinous neoplasms (IPMNs) and mucinous cystic neoplasms (MCNs) carry malignant potential and therefore require surveillance [39]. As a result, MRI surveillance plays an important role in monitoring PCLs for malignant transformation and guiding timely intervention. 

Contrast-enhanced MRI with magnetic resonance cholangiopancreatography (MRCP) is the current standard for PCL assessment, particularly for IPMNs. No definite MRI protocol can be recommended for the diagnosis or surveillance of patients with PCN because of the heterogeneity of the published data and the lack of dedicated comparative studies [40]. However, C-MRI would typically include breath-hold axial in- and out-of-phase T1-weighted (T1W) gradient-recalled echo (GRE) to evaluate for intravoxel fat, axial and coronal T2-weighted single-shot FSE and heavily T2-weighted 2D/3D MRCP to visualize the internal architecture and relationship of the PCL to the main pancreatic and main duct caliber and/or presence of abrupt duct change in diameter, and dynamic fat-suppressed 3D T1-weighted GRE acquired before and after IV contrast to assess for background pancreatic parenchymal signal intensity, presence of small solid lesions, and enhancement of mural nodules and the cyst wall [41].

C-MRI examinations are of variable length depending on the specific protocol chosen but are generally time-intensive, particularly if IV contrast is used. Repeated use of IV contrast also raises concerns about gadolinium deposition in the brain, kidney and bone [42]. In response, non-contrast A-MRI protocols have emerged as a faster and lower-cost alternative for surveillance of incidentally discovered PCLs. A-MRI protocols are variable but often include T1-weighted gradient echo and T2-weighted single-shot FSE, with or without 2D/3D MRCP, and with or without DWI [41].

#### 3.2.2. Potential Advantages of Abbreviated Pancreatic MRI Protocols

The primary advantage of A-MRI is reduction in scan time by removing the contrast-enhanced sequences. A few small, retrospective studies have compared C-MRI and A-MRI. Nougaret et al. (retrospective, *n* = 301) compared a C-MRI protocol of axial and coronal T2W, MRCP and pre- and post-contrast fat-suppressed T1W sequences to a retrospective A-MRI, which was the same except for the post-gadolinium-enhanced sequences. They found that lesion assessment was discordant between contrast-enhanced and non-contrast protocols in only 4.6% of cases and risk assignment into benign, indeterminate and malignant categories showed excellent inter-reader agreement for both A-MRI and C-MRI [43]. In a retrospective study of 123 subjects with a simulated A-MRI of coronal and axial heavily T2W, unenhanced T1W, and BH-3D-MRCP, Kang et al. showed A-MRI had excellent inter-reader agreement for categorizing IPMN into negative, indeterminate and suspicious for malignancy using pre-determined criteria [44]. There was good to excellent inter-reader agreement for main duct diameter and cystic size, but only moderate agreement for abrupt change in main duct diameter, which is an imaging feature of substantial clinical importance. Among a small number pathologically confirmed mural nodules (*n* = 29), the sensitivity for mural nodule detection in A-MRI was comparable to that in cMRI; however, evaluation is limited by a small sample size [44]. DWI and 3D MRCP sequences were not included in the A-MRI [44]. Johansson et al. reported that an ultra-shortened A-MRI protocol consisting of T2-weighted HASTE axial and 3D MRCP achieved comparable inter-reader agreement to C-MRI in 183 subjects [45], but in this study, the C-MRI group included two distinct protocols: a full exam with gadolinium as well as a longer, non-contrast enhanced exam, confounding interpretation. Additionally, 53/183 subjects were excluded because of insufficient image quality, potentially introducing bias. Kierans et al. (*n* = 87) found similar inter-reader agreement for worrisome features/high risk stigmata between C-MRI and A-MRI of 0.72 and 0.73 for reader 1 and 2, respectively [46]. A-MRI was defined as unenhanced T1-, T2-weighted, and DWI+/− 3D MRCP and differed from C-MRI only by exclusion of the dynamic post-gadolinium-enhanced images. IV contrast did not significantly affect differentiation between benign and malignant PCLs, with identical AUC values of 0.622 [95% CI 0.44, 0.74] (*p* = 1.0) for reader 1 and similar AUC values of 0.569 [95% CI 0.39, 0.71] and 0.589 [95% CI 0.40, 0.72] (*p* = 0.08) for reader 2 between the non-contrast and contrast-enhanced datasets when the presence of any worrisome feature/high-risk stigmata was considered positive; pathologic resection or cytopathology served as the reference standard [46]. Although the AUC values are similar with and without contrast, confidence intervals are wide and the authors state that the study is underpowered to detect a difference in diagnostic accuracy between C-MRI and A-MRI.

Efficiency is reported as a key advantage of A-MRI, particularly for long-term surveillance programs. Abbreviated protocols have reportedly shortened examination time by approximately half (5–15 min vs. 30–40 min). Estimates of time reduction are highly variable because of differences in A-MRI protocol definitions, which range from identical to C-MRI except for contrast-enhanced sequences to ultrashort protocols consisting of only T2W and 3D MRCP [39,43,45]. Potential cost savings are another proposed benefit of A-MRI [39]; however, systematic cost–benefit analyses have not been performed. Reported cost analyses are highly variable with respect to input parameters, and assumptions are very dependent on the specific health care system modeled, and not generalizable given the small sample sizes.

Optimal sequence selection remains an area of ongoing investigation, with lack of a standard definition for A-MRI. Malekzadeh et al. (retrospective, *n* = 114) found that both contrast-enhanced sequences and DWI added no benefit for detecting Branch-Duct IPMN degenerative features (lesion size, cyst growth, presence of mural nodule, and increased main pancreatic duct diameter) [47]. Excluding DWI reduced the exam time by 4 min [47]. 

Other studies have reported potential benefits of DWI [41] Fukuba et al. (*n* = 217) demonstrated that DWI alone achieved a specificity of 94.5% and sensitivity of 94.4% compared with 94.5% and 100% when combined with MRCP [48]. The authors highlighted its potential value in high-risk patients without dilation of the caudal pancreatic duct or with suspected pancreatic tumors [48]. Other studies, including those by Maio et al., Crippa et al. and Kim et al. support the inclusion of DWI for IPMN screening and malignancy prediction [49,50,51]. Some studies suggest T2W and DWI can achieve diagnostic accuracy comparable to C-MRI [49,50,51].

#### 3.2.3. Limitations of Abbreviated Pancreatic MRI Protocols

Support for IV contrast for surveillance MRI stems from its potential to improve sensitivity for detecting malignant transformation in PCLs. Pozzi-Mucelli et al. and Nougaret et al. emphasize its importance in differentiating mucous plugs from true mural nodules [39,43].

Additional proposed benefits include improved assessment of cyst wall enhancement, although reproducibility and inter-reader agreement remain poor [52]. IV contrast may also facilitate detection of solid pancreatic lesions, such as pancreatic ductal adenocarcinoma, which may occur more frequently in patients with PCLs, as well as improved identification of mural nodules and invasive carcinoma [53,54]. Accordingly, in higher-risk clinical contexts, contrast-enhanced MRI is recommended by the American College of Radiology [54].

Reader experience is another consideration, although minimal literature exists in the context of PCLs. D’Onofrio et al. reported that A-MRI can be adopted for experienced radiologists, but those more junior or working in low-volume centers should rely on C-MRI protocols for IPMN surveillance based on differences intra-observer agreement and concordance of radiologist impression with clinical decisions for readers of differing experience levels [55]. Because this suggestion is based on only three radiologists, it cannot be generalized to other settings.

#### 3.2.4. Guidelines–Pancreatic MRI Protocols

The Korean Society of Abdominal Radiology (KSAR) suggests that IV contrast can be omitted for serial follow-up of incidental PCLs [56]. The Society of Abdominal Radiology Disease Focused Panel (SAR-DFP) similarly supports non-contrast surveillance in low-risk patients (no main pancreatic duct dilation, mural nodularity or other worrisome features/high risk-stigmata, no symptoms, family history, or genetic predisposition) [41]. However, SAR-DFP notes that IV contrast enables a more comprehensive assessment, including evaluation of the complete pancreas and detection of cancers not directly related to cysts, an important consideration given the elevated pancreatic cancer risk in patients with PCLs [41].

The Canadian Association of Radiologists endorses contrast-enhanced MR pancreas with MRCP within 1 year of PCL identification for patients aged 50–75 years, then a limited pancreatic cyst follow-up MRI every 2 years up to 5 years or age 75, though specific sequences for the limited pancreatic MRI are not explicitly listed [57]. The American College of Radiology Incidental Findings Committee acknowledges that the routine use of IV contrast for follow-up MRI of pancreatic cysts is controversial [58]. While A-MRI offers shorter scan times and reduced cost, IV contrast may still provide benefit in detecting mural nodule enhancement and PDAC [58]. Overall, the guidelines favor use of IV contrast for initial characterization and in high-risk patients, but ultimately the heterogeneity of A-MRI protocols in the literature limits comparison of the reported performance metrics and precludes recommendation of any single C-MRI or A-MRI protocol.

#### 3.2.5. Discussion of Abbreviated Pancreatic MRI Protocols

Interest in A-MRI for low-risk surveillance is driven by the relatively indolent nature of smaller PCLs, large number of patients potentially requiring surveillance, shortened exam times, and removal of risks and costs associated with IV access and contrast administration. The current literature is limited by small sample size, retrospective analysis, lack of standardization of A-MRI definition, and variability in reference standards. This heterogeneity makes direct comparisons difficult and limits generalizability. Additionally, most studies are single-center and focus on endpoints such as inter-reader agreement rather than malignant transformation confirmed by histopathology or survival outcomes [46,59]. Such surrogate endpoints are less relevant if they do not evaluate features that predict malignancy or alter clinical decision making. In general, reducing scan time by removing the contrast-enhanced sequences may provide the most tangible benefit of A-MRI; however, the specific amount of time saved is difficult to quantify because sequence acquisition times only are reported, without consideration of time required for IV placement, patient positioning and set-up in the magnet bore, or repeat sequences due to patient motion or discomfort, which may be greater contributors to overall MRI exam length than any one sequence alone.

There is potential for A-MRI to support sustainable, longitudinal surveillance of low-risk pancreatic cystic lesions within cancer prevention pathways. However, data remain limited, protocols are heterogeneous, and most guidelines favor contrast-enhanced MRI [41]. A reasonable approach may be to use C-MRI for baseline assessment, followed by surveillance with an A-MRI protocol that excludes post-contrast imaging, but retains additional sequences, including DWI. Consideration could be given to a recall system for IV contrast, like what has been proposed for prostate imaging [25,26], if certain pre-defined features such as lesion growth, development of a mural nodule, or abrupt pancreatic duct cut-off develop on surveillance scans.

Given the heterogeneity of A-MRI protocols used across these studies, the reported performance metrics are not directly comparable and the generalizability of these findings to any single A-MRI configuration should be interpreted with caution. Prospective, multi-center studies are needed to clarify optimal sequences, reader performance, and cost-effectiveness [60].

### 3.3. Liver

#### 3.3.1. Background

Hepatocellular carcinoma (HCC) is the fourth leading cause of cancer-related mortality worldwide [61]. Semiannual ultrasound (US) with or without alpha-fetoprotein (AFP) is recommended for HCC surveillance by several societies, including the American Association for the Study of Liver Disease (AASLD), the American Gastroenterological Association (AGA), and the Asian Pacific Association for the Study of Liver (APASL) [11,62,63]. Target populations include patients with cirrhosis of any etiology and select non-cirrhotic individuals with chronic Hepatitis B who would be candidates for curative treatment. The sensitivity of US with AFP for early-stage HCC detection is limited to approximately 61% and may be further reduced in patients with obesity [11]. Much of the evidence supporting semi-annual US surveillance is derived from hepatitis B predominant populations [64,65]. However, in Western populations metabolic dysfunction-associated steatotic liver disease (MASLD) has become a leading cause of chronic liver disease [66]. MASLD is associated with obesity, which can degrade US image quality and limit the effectiveness of US-based surveillance [66]. 

C-MRI has demonstrated higher sensitivity for HCC detection compared to US; however, its role as a routine surveillance tool remains limited by cost, scanner availability, examination time, and patient preference [11]. A-MRI has been proposed as a potential alternative, offering shorter acquisition times (approximately 12–15 min vs. 40 min for C-MRI) and potentially lower cost in high-risk populations such as patients with cirrhosis or suboptimal US visualization [67,68,69]. Importantly, A-MRI is not intended to replace US in low-risk populations, where US remains effective and cost-effective [69].

Several A-MRI strategies have been described, including non-contrast protocols (T1, T2, DWI), hepatobiliary-phase protocols (post-hepatocyte-specific contrast) and dynamic contrast-enhanced protocols [70,71]. Non-contrast A-MRI avoids the risks and cost associated with IV contrast, whereas contrast-enhanced protocols enable evaluation of Liver Imaging Reporting and Data Systems (LI-RADS) major features, including arterial phase hyperenhancement, venous-phase washout, and enhancing capsule [67]. As eligibility for curative therapies is often stage-dependent, even modest improvements in early detection could improve clinical outcomes [71,72,73]. 

#### 3.3.2. Potential Advantages of Abbreviated Liver MRI Protocols

Available studies suggest that A-MRI may demonstrate diagnostic performance comparable to C-MRI for HCC detection, although the literature is heterogeneous with respect to protocol design, study populations, and reference standards. In a meta-analysis of 7 studies, Kim et al. reported comparable pooled sensitivity and specificity between various unenhanced abbreviated protocols and C-MRI (87% and 94% vs. 84% and 94%, respectively) [69]. Khatri et al. evaluated a dynamic contrast-enhanced A-MRI protocol in 93 patients using an equivalence design with five independent readers and demonstrated equivalence to C-MRI in sensitivity, specificity, and accuracy within a predefined margin of ±0.05, though limited by a relatively small sample size [67]. Notably, most studies comparing A-MRI to C-MRI were not conducted in true surveillance settings, which may overestimate real-world performance. 

A subset of the literature directly compares unenhanced and contrast-enhanced A-MRI protocols for HCC surveillance. In a meta-analysis of 27 studies, Maung et al. reported pooled sensitivity and specificity of 83% and 91% for unenhanced A-MRI (21 studies) compared with 88% and 94% for contrast-enhanced A-MRI (15 studies) [74]. While the difference in sensitivity did not reach statistical significance (*p* = 0.078), the 5-percentage point gap may be clinically meaningful in a surveillance population where the goal is to detect small, early-stage HCC. Gupta et al. (15 studies, 2807 patients) reported pooled per-patient sensitivity and specificity of 86% and 94% for unenhanced protocols (11 studies) vs. 87% and 94% for contrast-enhanced protocols (7 studies), with no significant difference [75]. A-MRI performance was consistent across subgroups regardless of study design, screening setting, and underlying liver disease, although subgroup analyses were limited by small sample sizes and study heterogeneity [75]. 

Several patient and disease characteristics further influence A-MRI diagnostic performance. Maung et al. found significantly higher sensitivity in patients with chronic hepatitis B compared with other etiologies (87% vs. 78%, *p* = 0.003), with meta-regression identifying hepatitis B prevalence and geographic region (Eastern vs. Western countries) as major sources of heterogeneity [74]. Beyond intrinsic disease characteristics, US image quality itself is a recognized limitation of current surveillance, particularly in patients with obesity. Vithayathil et al. evaluated unenhanced A-MRI specifically in patients with inadequate or suboptimal US examinations, including those with obesity, and found adequate liver visualization in 97% of patients, with detection of additional lesions not seen on US [76]. 

The feasibility of A-MRI as a surveillance tool depends on its cost effectiveness. A cost-utility analysis found unenhanced A-MRI to be more cost-effective than US with AFP in both low- and high-income healthcare settings, with incremental cost-effectiveness ratios (ICER) of $3667/QALY (quality-adjusted life years) in Thailand and $37,062/QALY in the United States [77]. In a Markov decision-analytic model, Goossens et al. found that risk-stratified surveillance using A-MRI for intermediate and high-risk patients with cirrhosis, while foregoing screening in low-risk patients, was cost-effective compared with non-stratified biannual ultrasound, with an ICER of $2100/QALY [78]. Kim et al. similarly reported that semiannual MRI had an ICER of $25,202/QALY at an annual HCC incidence of 3%, becoming cost-effective when annual incidence exceeded 1.81% [79]. However, these estimates are highly sensitive to assumptions about HCC incidence, imaging costs, and US quality. Both the Goossens and Kim models were based predominantly on hepatitis B-associated cirrhosis populations that may not be generalizable to settings where metabolic or alcohol-related liver disease predominates. 

#### 3.3.3. Limitations of Abbreviated Liver MRI Protocols

A-MRI demonstrates reduced sensitivity for small, early-stage HCC, although this limitation is also observed with C-MRI and US. In a meta-analysis of seven studies (1830 patients), Kim et al. found that pooled sensitivity was lower for very early-stage HCC, defined as HCC ≤ 2 cm, (77%) compared with any stage HCC (85%) [69]. Gupta et al. similarly reported a per-lesion sensitivity of 69% for HCC < 2 cm compared with 86% for HCC ≥ 2 cm [75]. Girardet et al. found that unenhanced A-MRI sensitivity for HCCs ≤ 2 cm was only 52.9%, which was significantly lower than contrast-enhanced A-MRI (85.3%) and C-MRI (88.2%). However, when AFP was added, sensitivity improved to 88.2%, which was equivalent to both contrast-enhanced protocols [80]. Biomarker augmentation may partially compensate for the limitations of unenhanced A-MRI in detecting small lesions, which is consistent with the AASLD recommendation that serum biomarkers may incrementally improve the performance of imaging-based surveillance [11]. 

Not all A-MRI protocols are equivalent, and protocol selection may meaningfully influence diagnostic reliability. In a meta-analysis of eight studies, Kim et al. found that DWI-only protocols had the lowest inter-reader agreement, with a pooled kappa coefficient of 0.64 compared with 0.80 for contrast-enhanced A-MRI and 0.98 for HBP-A-MRI, suggesting that sequence composition may impact interpretive consistency, although findings were limited by study heterogeneity [81]. Maung et al. reported that unenhanced approaches occasionally missed lesions due to comparatively lower contrast-to-noise ratio on T1 and T2-weighted sequences, though chemical-shift imaging may partially mitigate this limitation [74]. These findings underscore that not all A-MRI protocols are equivalent, and that protocol selection may meaningfully influence diagnostic reliability. 

IV contrast administration introduces several practical limitations in a surveillance setting. Although Preethi et al. reported equivalent per-lesion sensitivity and NPV of 100% for lesions > 10 mm with both non-contrast and contrast-enhanced A-MRI, these results should be interpreted cautiously given the small sample size [82]. The use of IV contrast may restrict use in patients with renal impairment and can increase exam time and cost [83]. In addition, concerns have been raised regarding gadolinium deposition in the brain, bone, kidney and liver with repeated exposure. HPB-A-MRI, which utilizes gadoxetic acid, is associated with substantially higher costs compared to extracellular gadolinium-based agents. Furthermore, its diagnostic performance may be reduced in cirrhotic patients, as hepatic uptake of gadoxetic acid relies on functioning hepatocyte transporters which are often impaired in this population [84]. 

While preceding analyses suggest A-MRI may be a cost-effective surveillance strategy in higher-risk populations, not all economic evaluations reach the same conclusion. A Singapore-based cost-effectiveness analysis compared US and unenhanced A-MRI (T2, DWI) for HCC surveillance in a simulated cohort of 482,000 at-risk patients over 40 years [85]. US resulted in substantial QALY gains compared with no surveillance (ICER of approximately $1193/QALY), while non-contrast A-MRI resulted in only marginal additional benefit at higher cost (ICER of approximately $9720/QALY) [85]. Despite its superior diagnostic accuracy, unenhanced A-MRI was less cost-effective than US for this general at-risk population. These results likely reflect local factors: US is relatively inexpensive in Singapore, and the at-risk population is predominately hepatitis B-related, with higher baseline HCC incidence [85]. In contrast, North American populations have higher rates of MASLD, often associated with larger body habitus and reduced US sensitivity [85,86]. These differences underscore that the cost-effectiveness of A-MRI is context-dependent and should not be extrapolated across healthcare systems without local validation [85]. 

#### 3.3.4. Guidelines–Liver MRI Protocols

Society guidelines provide a framework for integrating A-MRI into HCC surveillance, though recommendations remain cautious. The AASLD recommends US with AFP as the primary surveillance strategy for HCC but supports the use of CT or MRI in patients with an inadequate US [87]. Specifically, the AASLD provides a conditional recommendation based on low-certainty evidence (level 3) for the consideration of contrast-enhanced MRI for HCC surveillance in patients where US-based surveillance is suboptimal [11]. The AASLD acknowledged that US may be unreliable in patient populations with truncal obesity or marked parenchymal heterogeneity and encourages further studies to better clarify the population best suited for cost-effective and safe use of CT or MRI-based surveillance [11].

The LI-RADS US surveillance v2024 Core provides additional guidance for patients with severely limited US visualization (categorized as VIS-C). In patients without risk factors for repeat VIS-C (such as MASH or alcohol-related cirrhosis; Child-Turcotte-Pugh class B or C cirrhosis; BMI ≥ 35 kg/m^2^), US should be repeated in 3 months. If the repeat US remains VIS-C, alternative surveillance strategies such as C-MRI, A-MRI or multiphase CT should be considered instead of repeat US [88]. The LI-RADS US surveillance v2024 Core further states that in patients with risk factors for repeat VIS-C, alternative surveillance strategies should be considered instead of repeat US. The choice of alternative surveillance strategy should be tailored to the patient and take into account local preferences, expertise, resources, and cost-effectiveness considerations discussed above [88]. In a recent clinical practice update, the AGA briefly addressed that A-MRI shows promise for surveillance of HCC yet does not provide any explicit recommendations. They do, however, state that results of the Preventing Liver Cancer Mortality Through Imaging with Ultrasound vs. MRI (PREMIUM) trial, a large study based in the United States, will hopefully provide important insight into A-MRI’s utility for HCC surveillance [62]. 

#### 3.3.5. Discussion of Abbreviated Liver MRI Protocols

The A-MRI literature for HCC surveillance has largely focused on comparisons between A-MRI and US, as well as A-MRI and C-MRI. Although more recent work has begun to explore optimal A-MRI sequences for specific patient populations and to compare diagnostic performance across protocol variants, several important gaps remain. Table 1 outlines the potential advantages and disadvantages associated with various A-MRI protocols considered for HCC surveillance, including non-contrast, hepatobiliary phase-enhanced, and dynamic contrast-enhanced approaches.

Relatively few studies have been conducted in true surveillance settings that report the annual HCC incidence of the populations studied. The diagnostic performance of A-MRI reported in prospective studies appears comparable to that observed in retrospective analyses, but the limited number of prospective studies restricts the strength of this observation [67,75]. Future prospective studies conducted in true surveillance populations would better validate diagnostic performance, help determine optimal surveillance intervals, and provide insight into clinical outcomes. The AMRIUS trial, a randomized controlled study of 806 high-risk patients with cirrhosis evaluating A-MRI versus US for HCC surveillance, along with the PREMIUM trial, represent important steps in this direction [89,90].

Geographic and etiologic differences also limit direct comparison between studies. Many A-MRI studies have been conducted in cohorts with a predominance of hepatitis B virus infection, which differs from the higher prevalence of MASLD in Western populations [75]. Many studies also do not stratify results by ultrasound quality or specifically include patients in which ultrasound was suboptimal, which complicates the evaluation of risk-adapted surveillance strategies using A-MRI.

For A-MRI to be integrated into a feasible surveillance strategy, further cost-effectiveness analyses are needed that account for several key variables. Specifically, consideration for variability in disease populations, differences between private and public healthcare systems, and resources availability. 

Overall, A-MRI represents a potentially important evolution in HCC surveillance for high-risk patients. By improving lesion detection in select at-risk populations, A-MRI may support earlier stage diagnosis and better alignment with curative treatment pathways. 

## 4. Conclusions

A-MRI has emerged as a potential approach to improve the accessibility and efficiency of MRI within cancer-detection and surveillance pathways, particularly in prostate cancer detection and surveillance, pancreatic cystic lesion surveillance and hepatocellular carcinoma surveillance. A-MRI may also facilitate reduced cost and contrast utilization in MRI-based surveillance programs.

The performance and clinical utility of A-MRI are influenced by factors such as disease prevalence, lesion characteristics, imaging indication, reader experience, and local practice environments. In the prostate, bpMRI, which omits DCE, may achieve similar diagnostic performance to mpMRI in selected treatment-naïve patients. However, DCE remains valuable for risk stratification of equivocal PI-RADS 3 peripheral zone lesions and in settings of poor image quality due to artifacts. In the pancreas, non-contrast A-MRI may facilitate comparable assessment of low-risk pancreatic cystic lesions compared to C-MRI in selected surveillance populations. Contrast-enhanced imaging may still improve evaluation of mural nodules, cyst wall enhancement and associated pancreatic malignancy. In the liver, A-MRI may offer improved sensitivity compared to US surveillance, particularly in patients with limited US visualization. Both non-contrast and contrast-enhanced A-MRI protocols demonstrate higher reported sensitivity than US for HCC detection in high-risk patients. However, cost-effectiveness is context-dependent and may vary substantially between healthcare systems and patient populations. The key findings of this review are summarized further in Table 2.

Several limitations of the available literature should be acknowledged. The heterogeneity in A-MRI protocols and the predominance of single-center retrospective studies limit direct comparison and generalizability. Future prospective multicenter studies using standardized protocols and evaluation in true surveillance settings will be important to better define the potential role of A-MRI in surveillance pathways. Additional cost-effectiveness analysis that account for local practice environments and healthcare systems will also help inform local adoption and guideline recommendations.

In its current state, A-MRI may be best positioned as a complementary, context dependent strategy that could support more efficient use of MRI resources in select settings, rather than a universal replacement for C-MRI. 

## Figures and Tables

**Table 1:** Potential advantages and disadvantages for abbreviated MRI protocols for HCC surveillance.

A-MRI Protocol for HCC Surveillance	Potential Advantages	Potential Disadvantages
Non-contrast (T1, T2, DWI)	IV gadolinium not needed Reduced in-bore time compared with complete protocol May be more sensitive than ultrasound in selected populations	Less sensitive than complete protocol Relies on LI-RADS ancillary features for detection; call back for DCE required if surveillance scan is positive
T2W and DWI plus hepatobiliary phase; dynamic contrast-enhanced sequences not acquired	Reduced in-bore time compared with complete protocol May be more sensitive and specific than ultrasound in selected populations LI-RADS ancillary features based on T2 and DWI can be assessed	IV gadolinium required increased cost of gadoxetic acid compared to extracellular gadolinium-based agents Relies primarily on LI-RADS ancillary features (the LI-RADS major features non-rim arterial phase hyperenhancement, capsule and washout cannot be assessed); call back required Diagnostic performance may be reduced in patients with cirrhosis
Dynamic contrast-enhanced (DCE) sequence with an extracellular gadolinium-based agent	Reduced in-bore time compared with complete protocol May be more sensitive and specific than ultrasound in selected populations Enables the use of LI-RADS major features that require DCE (non-rim arterial phase hyperenhancement, capsule, and washout) which permits the of LI-RADS 5 in lesions > 10 mm if criteria are met	IV gadolinium required Cannot use ancillary LI-RADS features based on T2 or diffusion-weighted imaging

**Table 2:** Summary of the clinical indications, potential advantages and limitations for abbreviated MRI in the prostate, pancreas, and liver. This table represents a simplified synthesis and should be interpreted in the context of the heterogeneity discussed in the text.

Organ System	Oncologic Role	Abbreviated MRI Protocol (Typical Sequences)	Integration into Cancer Care Pathways	Potential Advantages	Key Limitations
Prostate	Detection of clinically significant prostate cancer; active surveillance; biopsy triage	T2-weighted imaging and diffusion-weighted imaging (bp MRI); no dynamic contrast-enhanced imaging	May improve access to MRI within biopsy-triage pathways; supports risk adapted use of DCE	Shorter acquisition time; elimination of intravenous contrast; comparable sensitivity and NPV to mpMRI; improved scanner throughput	Limited incremental information for equivocal PI-RADS 3 lesions; potential reliance on reader experience; not uniformly endorsed by guidelines
Pancreas	Surveillance of pancreatic cystic lesions with malignant potential	T2-weighted imaging; T1-weighted gradient-echo; ±MRCP; ±diffusion-weighted imaging; no intravenous contrast	May enable sustainable long-term surveillance programs; reduces repeated IV contrast exposure	Reduced scan time and cost; avoidance of repeated contrast exposure; comparable inter-reader agreement to C-MRI in low-risk patients	Reduced sensitivity for mural nodules and concomitant pancreatic cancer; protocol heterogeneity; limited data in high-risk populations
Liver	Surveillance for early hepatocellular carcinoma detection	Non-contrast (T1-weighted, T2-weighted, diffusion-weighted imaging) or contrast-enhanced abbreviated protocols	May enhance detection in patients with suboptimal US; supports risk-stratified surveillance models	Higher sensitivity than ultrasound; shorter exam time than C-MRI; potential cost-effectiveness in risk-stratified populations	Reduced sensitivity for lesions < 2 cm; inter-reader variability; cost-effectiveness dependent on population and healthcare setting

## Data Availability

No new data were created or analyzed in this study.

## Supplementary Materials

The following supporting information can be downloaded at: https://www.mdpi.com/article/10.3390/curroncol33060306/s1, Table S1: Checklist Items; Table S2: Ovid MEDLINE and Embase Search Strategy for the prostate, pancreas and liver.

## Abbreviations

The following abbreviations are used in this manuscript:

A-MRI	Abbreviated MRI
AASLD	American Association for the Study of Liver Diseases
AFP	Alpha-fetoprotein
bpMRI	Biparametric MRI
C-MRI	Complete MRI
csPCa	Clinically significant prostate cancer
DCE	Dynamic contrast-enhanced imaging
DWI	Diffusion-weighted imaging
GRE	Gradient-recalled echo
HCC	Hepatocellular carcinoma
HPB-A-MRI	Hepatobiliary phase abbreviated MRI
ICER	Incremental cost-effectiveness ratio
IPMN	Intraductal papillary mucinous neoplasm
IV	Intravenous
LI-RADS	Liver Imaging Reporting and Data System
MASLD	Metabolic dysfunction-associated steatotic liver disease
MCN	Mucinous cystic neoplasm
mpMRI	Multiparametric MRI
MRCP	Magnetic resonance cholangiopancreatography
NCCN	National Comprehensive Cancer Network
NPV	Negative predictive value
PCa	Prostate cancer
PCL	Pancreatic cystic lesion
PI-RADS	Prostate Imaging Reporting and Data System
PPV	Positive predictive value
PZ	Peripheral zone
QALY	Quality-adjusted life year
SAR-DFP	Society of Abdominal Radiology Disease Focused Panel
T1W	T1-weighted
T2WI	T2-weighted imaging
TRUS	Transrectal ultrasound
US	Ultrasound
