# Supplementary material for: Cancer Detection and Surveillance Pathways with Abbreviated MRI in the Prostate, Pancreas, and Liver: A Risk-Stratified Narrative Review
Source: Curr Oncol. 2026 May 24;33(6):306. doi: 10.3390/curroncol33060306 (PMC13298129; doi:10.3390/curroncol33060306)
Supplement: Supplementary file 1 [file curroncol-33-00306-s001.zip › curroncol-4248061-supplementary.pdf]

Supplementary Materials:

Table S1: Checklist Items

Section/topic	#	Checklist item	Reported on page or line #
**TITLE**			
Title	1	Identify the report as a Narrative Review.	1
**ABSTRACT**			
Unstructured summary	2	Provide an unstructured summary including, as applicable: background, objective, brief summary of narrative review and implications for future research, and clinical practice or policy development.	1
**INTRODUCTION**			
Rational/background	3	Describe the rationale for the review in the context of what is already known.	2
Objectives	4	Specify the key question(s) for the review topic.	2
**METHODS**			
Research selection	5	Specify the process for identifying the literature search (e.g. years considered, language, publication status, study design, and database of coverage.	3
**DISCUSSION/SUMMARY**			
Narrative	6	Discuss 1) research reviewed including fundamental or key findings, 2) limitations and/or quality of research reviewed, and 3) need for future research.	3-14
Summary	7	Provide an overall interpretation of the narrative review in the context of clinical practice for health professionals, policy development and implementations, or future research.	14-15

Table S2: Ovid MEDLINE and Embase Search Strategy for the prostate, pancreas and liver

Prostate	Ovid Medline – Total Results 417<br><br>1. (“Multiparametric MRI” or “mpMRI” or MR* or “Dynamic contrasted-enhanced imaging” or “Magnetic resonance imaging” or “DCE MRI” or “Dynamic contrast-enhanced imaging (DCE)”).ti,ab. or Multiparametric Magnetic Resonance Imaging/ or Diffusion Magnetic Resonance Imaging/ or Magnetic Resonance Imaging/<br>2. (“Prostate cancer” or “PCa” or “Clinically significant prostate cancer” or “csPCa” or prostate).ti,ab. or Prostatic Neosplasms/<br>3. (“Shortened” or “Reduced” or “Biparametric” or “bpMRI” or “bp-MRI” or “Biparametric magnetic resonance imaging” or “Abbreviated bi-parametric MRI” or “a-bpMRI” or “abbreviated MRI” or “Accelerated Biparametric MR Imaging” or “Accelerated imaging” or “Accelerated MRI”).ti,ab.<br>4. (“Diagnostic” or “Diagnostic accuracy”).ti,ab.<br>5. 1 and 2 and 3 and 4<br>6. Limit 5 to (english language and yr = “2015-Current”)<br><br>Embase – Total Results 1017<br><br>1. (“Multiparametric MRI” or “mpMRI” or MR* or “Dynamic contrasted-enhanced imaging” or “Magnetic resonance imaging” or “DCE MRI” or “Dynamic contrast-enhanced imaging (DCE)”).ti,ab. or Multiparametric Magnetic Resonance Imaging/ or Diffusion Magnetic Resonance Imaging/<br>2. (“Prostate cancer” or “PCa” or “Clinically significant prostate cancer” or “csPCa” or prostate).ti,ab. or prostate biopsy/ or prostatectomy/<br>3. (“Shortened” or “Reduced” or “Biparametric” or “bpMRI” or “bp-MRI” or “Biparametric magnetic resonance imaging” or “Abbreviated bi-parametric MRI” or “a-bpMRI” or “abbreviated

	MRI" or "Accelerated Biparametric MR Imaging" or "Accelerated imaging" or "Accelerated MRI").ti,ab. or Dynamic contrast-enhanced magnetic resonance imaging/ or Biparametric magnetic resonance imaging/<br>4. ("Diagnostic" or "Diagnostic accuracy").ti,ab. or diagnostic value/ or image quality/<br>5. 1 and 2 and 3 and 4<br>6. Limit 5 to (english language and yr = "2015-Current")
Pancreas	OVID-Medline – total results 144<br>1. Pancreatic Neoplasm/<br>2. ((Pancre* or intraductal papillary mucinous) adj3 (cancer or neoplasm or carcinoma or tumor or malignant or lesion*)).ab.kf,ti.<br>3. 1 or 2<br>4. ((MRI or sequence* or MR) adj3 (shortened or reduced or entire or Accelerated or Pancreatobiliary or Abbreviated or Non-contrast)).ab,kf,ti.<br>5. ((MRI or sequence* or MR) adj3 ((Without adj2 contrast) or (Contrast adj2 unenhanced) or (contrast adj2 enhanced))).ab,kf,ti.<br>6. Cholangiopancreatography, magnetic resonance/ or dynamic contrast enhanced magnetic resonance imaging/<br>7. 4 or 5 or 6<br>8. 3 and 7<br>9. (surveillan* or screen* or monitor* or observant* or (diagnostic adj2 accuracy)).ab,kf,ti.<br>10. 8 and 9<br>Embase – total results 250<br>1. Exp intraductal papillary mucinous tumor/<br>2. Exp pancreas cancer/<br>3. Exp pancreas cyst/

	4. Exp pancreas islet cell tumor/<br>5. ((Pancre* or intraductal papillary mucinous) adj3 (cancer or neoplasm or carcinoma or tumor or tumour or malignant or lesion*)). ab,kf,ti.<br>6. Or/1-5<br>7. ((MRI or sequence* or MR) adj3 (shortened or reduced or entire or Accelerated of Pancreatobiliary or Abbreviated or Non-contrast)).ab,kf,ti.<br>8. ((MRII or sequence* or MR) adj3 ((Without adj2 contrast) or (Contrast adj2 unenhanced) or (contrast adj2 enhanced))).ab,kf,ti.<br>9. Magnetic resonance cholangiopancreatography/<br>10. 7 or 8 or 9<br>11. 6 and 10<br>12. (surveillan* or screen* or monitor* or observant* or (diagnostic adj2 accuracy)).ab,kf,ti.<br>13. 11 and 12<br>14. limit 13 to yr-"2015-Current"<br>15. limit 14 to "remove medline records"<br>16. limit 15 to English language
Liver	OVID-Medline – Total Results 125<br>1. (HCC or Hepatocellular carcinoma or Liver lesion or hepatic lesions or Liver cancer).mp or exp Carcinoma, Hepatocellular/ or exp Liver Neoplasms/<br>2. ((MRI or sequence* or MR) adj3 ((Without adj2 contrast) or (Contrast adj2 unenhanced) or (contrast adj2 enhanced))).ab,kf,ti. Or exp Magnetic Resonance Imaging/<br>3. Surveillance.mp.

	4. (detection or early-stage HCC or early-stage detection or Screening or screened or screening tool or early detection).mp or exp “Early Detection or Cancer”/ or exp Mass Screening/<br>5. 1 and 2 and 3 and 4<br>6. Limit 5 to (English language and yr=”2015-Current”)<br><br>Embase – total results 201<br>1. (shortened or reduced MRI protocol or abbreviated MRI or abbreviated or abbreviated magnetic resonance imaging or AMRI or A-MRI or abbreviated contrast-enhanced MRI protocols or abbreviated protocols).mp.<br>2. (HCC or Hepatocellular carcinoma or Liver lesion or hepatic lesions or Liver cancer).mp or exp liver cell carcinoma/ or exp liver tumor/<br>3. (complete multiphase MRI or magnetic resonance imaging or MRI or Full CE-MRI protocols or Standard dynamic contrast MRI or Full standard protocol or Complete protocol diagnostic MRI or cMRI or C-MRI).mp. or exp nuclear magnetic resonance imaging/ or exp dynamic contrast-enhanced magnetic resonance imaging/<br>4. Surveillance.mp. or exp follow up/<br>5. (early stage detection or (detect* or screening) or screened or screening tool).mp pr exp mass screening/ or exp early cancer diagnosis/ or exp cancer screening/<br>6. 1 and 2 and 3 and 4 and 5<br>7. Limit 6 to (English language and yr=”2015-Current”)
